# Treatment of Diphtheria

**Published:** 1879-05

**Authors:** 


The Treatment of Diphtheria.—D. D. Hovell writes to the *Lancet:*—The late Mr. John Scott used to treat severe cases of cynanche with a "scavenger"—viz, calomel, jalap, and scammony, after which they usually got well. Looking at a diphtheritic throat one day, I said to myself, Why should I not give this patient a scavenger? I did so, and remarked that the subsequent course of the disease was certainly cut short. I have since adopted the plan, and invariably found that the same good results have followed; the tendency to fresh deposits has much diminished, and in many cases is wholly prevented. I can call to mind more than one case in which the attempt as a fresh deposit was clear, but very feeble. Unhappily I have seen a good deal of diphtheria at different times since its appearance in this country, about twenty-two years ago, and have treated it, on the whole not unsuccessfully, but it was about a year ago that the above circumstances almost accidentally forced the conviction on my mind that *elimination of the poison* ought to be the first object in treatment. How this elimination takes place through the bowels I know not, but diphtheria is a disease of blood poisoning, and there is no reason why the poison should not be eliminated through the bowels, any less or more than in enteric fever—in throat fever than in bowel fever. I cannot refuse the evidence of my experience, that since I have adopted the practice of purging at the outset the course of the disease has been invariably more favorable, nor the testimony of those who declare that the dejections thus produced are abominably offensive. At any rate, the practical effect of "the scavenger" recommends itself to my mind in a much higher degree than the scientific inaction of temporizing and waiting till the disease has run its course, however masterly such inaction may appear; or than the inferior, but by no means futile plan, of trying to neutralize the effects of the poison. Invaluable as the scientific knowledge is, it ought not to stop short, nor rest content, without improvement in practice. Elimination, then, (1) through the bowels, (2) through the kidneys. Chlorate of potash drinks should be given frequently, incessantly. Where the nose is affected, the same solution or that of permanganate of potash should be drawn up frequently through the nostrils, so as thoroughly to cleanse them. When the patient cannot swallow, nutrient enemata should be given four times a day; not too frequently if they are to be retained. For this, nothing is better than Dr. Munk's mixture, a tumbler full of milk gruel, a new-laid egg, a tablespoonful of Brandy.—*Western Lancet.*

